# Extracellular Signaling Molecules from Adipose-Derived Stem Cells and Ovarian Cancer Cells Induce a Hybrid Epithelial-Mesenchymal Phenotype in a Bidirectional Interaction

**DOI:** 10.3390/cells14050374

**Published:** 2025-03-04

**Authors:** Vinícius Augusto Simão, Juliana Ferreira Floriano, Roberta Carvalho Cesário, Karolina da Silva Tonon, Larissa Ragozo Cardoso de Oliveira, Flávia Karina Delella, Fausto Almeida, Lucilene Delazari dos Santos, Fábio Rodrigues Ferreira Seiva, Débora Aparecida Pires de Campos Zuccari, João Tadeu Ribeiro-Paes, Russel J. Reiter, Luiz Gustavo de Almeida Chuffa

**Affiliations:** 1 Department of Structural and Functional Biology, Institute of Biosciences, São Paulo State University (Unesp), Botucatu 18618-689, São Paulo, Brazil; 2 Bioengineering & Biomaterials Group, School of Pharmaceutical Sciences, São Paulo State University (Unesp), Km 01 Araraquara-Jaú Road, Araraquara 14800-903, São Paulo, Brazil; 3 Department of Biochemistry and Immunology, Ribeirao Preto Medical School, University of São Paulo (Usp), Ribeirão Preto 14049-900, São Paulo, Brazil; 4 Institute for Biotechnology, São Paulo State University (Unesp), Botucatu 18607-440, São Paulo, Brazil; 5 Department of Chemistry and Biochemistry, Institute of Biosciences, São Paulo State University (Unesp), Botucatu 18618-689, São Paulo, Brazil; 6 Department of Molecular Biology, Faculty of Medicine of São José do Rio Preto (Famerp), São José do Rio Preto 15090-000, São Paulo, Brazil; 7 Department of Biotechnology, School of Sciences, Humanities and Languages, São Paulo State University (Unesp), Assis 19806-900, São Paulo, Brazil; 8 Department of Cell Systems and Anatomy, UT Health, San Antonio, TX 78229, USA

**Keywords:** small extracellular vesicles, exosome, secretome, conditioned medium, adipose-derived mesenchymal stem cells, SKOV3, OVCAR3, indirect co-culture, cell invasion and migration, metastable phenotype

## Abstract

Ovarian cancer (OC) is characterized by high mortality rates due to late diagnosis, recurrence, and metastasis. Here, we show that extracellular signaling molecules secreted by adipose-derived mesenchymal stem cells (ASCs) and OC cells—either in the conditioned medium (CM) or within small extracellular vesicles (sEVs)—modulate cellular responses and drive OC progression. ASC-derived sEVs and CM secretome promoted OC cell colony formation, invasion, and migration while upregulating tumor-associated signaling pathways, including TGFβ/Smad, p38MAPK/ERK1/2, Wnt/β-catenin, and MMP-9. Additionally, OC-derived sEVs and CM induced a pro-tumorigenic phenotype in ASCs, enhancing their invasiveness and expression of tumor-associated factors. Notably, both ASCs and OC cells exhibited increased expression of E-cadherin and Snail/Slug proteins, key markers of epithelial/mesenchymal hybrid phenotype, enhancing cellular plasticity and metastatic potential. We also demonstrated that these cellular features are, at least in part, due to the presence of tumor-supportive molecules such as TNF-α, Tenascin-C, MMP-2, and SDF-1α in the CM secretome of ASCs and OC cells. In silico analyses linked these molecular changes to poor prognostic outcomes in OC patients. These findings highlight the critical role of sEVs and tumor/stem cell-derived secretome in OC progression through bidirectional interactions that impact cellular behavior and phenotypic transitions. We suggest that targeting EV-mediated communication could improve therapeutic strategies and patient outcomes.

## 1. Introduction

Ovarian cancer (OC) represents the second most lethal gynecological malignancy and the sixth leading cause of cancer deaths among women [1]. This high mortality rate is predominantly attributed to late-stage diagnosis, tumor recurrence, and metastatic progression [2]. The asymptomatic nature of early-stage disease results in 61% of patients being diagnosed at advanced stages (III and IV), often accompanied by extensive dissemination within the peritoneal cavity. In these cases, chemotherapy resistance associated with metastasis promotes a survival rate of only 20% [3,4]. Therefore, it is essential to investigate the mechanisms underlying relapse and metastasis to the development of innovative therapeutic approaches.

Studies have shown that adipose tissue plays a pivotal role in the progression of ovarian tumors [5,6,7,8]. In addition to providing structural support through fat layers adjacent to the primary epithelial mass of OC, adipose tissue acts as a source of stromal cells essential for tumor development. In response to tumor-derived signals, various cell types—including endothelial cells, fibroblasts, immune cells, and mesenchymal stem cells (MSCs)—are stimulated to proliferate and migrate toward the tumor microenvironment (TME), thereby enhancing metastatic activity [9,10]. Among the cell populations contributing to the ovarian TME, adipose-derived mesenchymal stem cells (ASCs) are subject to alterations in their plasticity, differentiating into malignant phenotypes that facilitate tumor progression through the secretion of proangiogenic and immunosuppressive factors [11,12]. Notably, several studies have reported the ability of OC cells to induce malignant differentiation in ASCs [13,14,15].

Extracellular vesicles (EVs) have recently been identified as critical mediators of intercellular communication, facilitating the transfer of genetic material, lipids, mitochondria, and proteins via lipid bilayer-enclosed particles to regulate cellular activities in recipient cells [16,17,18]. In the TME, EVs play a central role in cell signaling, transmitting diverse messages through autocrine and paracrine pathways. These EV-mediated signals can drive the differentiation of ASCs into tumorigenic phenotypes when originating from OC cell lines [19,20] or enhance tumor activity when derived from ASCs [2,21,22,23]. Conversely, other studies have demonstrated potential antitumor effects of ASC-derived EVs or their conditioned medium (CM), including the suppression of proliferation and the induction of apoptosis in OC cells [24,25,26,27]. This apparent dichotomy underscores the need for further research to elucidate the intricate bidirectional communication between ASCs and tumor cells in the context of OC.

In this study, we investigated the role of extracellular signaling molecules secreted by ASCs and OC cells, present either in the CM secretome or within small extracellular vesicles (sEVs; <200 nm), in modulating cellular responses in vitro under different culture conditions. Our findings clearly demonstrate the bidirectional capacity of both cell types to respond to external signals, primarily contributing to the redirection of cellular activity in favor of tumor progression. This phenomenon is mediated either through the malignant transformation of ASCs triggered by exposure to OC-derived factors or via the amplification of ovarian metastatic potential driven by ASC-derived extracellular mediators.

## 2. Materials and Methods

### 2.1. ASC Isolation, Culture, and Characterization

Adipose- derived MSCs (ASCs) were obtained from healthy female donors undergoing abdominoplasty procedures, with informed consent provided in accordance with the Research Ethics Committee of Botucatu School of Medicine, registered on the Plataforma Brasil (CAAE: 55033521.1.0000.5411). Patients were screened and excluded if they had a pre-existing cancer diagnosis. Additional exclusion criteria included a history of metabolic syndrome. ASCs were isolated as previously described [28,29] and used in experiments up to the 4th passage or cryopreserved at 196 °C. ASCs were cultured in 175 cm^2^ T-flasks in Dulbecco’s Modified Eagle’s medium (DMEM/Ham’s F-12) (BR30004-05, Nova Biotecnologia, Cotia, Brazil) supplemented with 10% fetal bovine serum (FBS) (10Bio500, Nova Biotecnologia, Cotia, Brazil) and 1% of antibiotic/antimycotic (AA) solution (13-30258-05, Nova Biotecnologia, Cotia, Brazil). Cultures were maintained in a humidified atmosphere incubator under standard conditions (37 °C and 5% CO_2_).

Prior to experimentation, 4.0 × 10^6^ ASCs were subjected to immunophenotypic characterization by flow cytometry using the FACSCanto II flow cytometer (Becton Dickinson, San Jose, CA, USA). The cells were incubated using the following antibodies: anti-CD34 (12-0349-42 PE), anti-CD45 (12-0459-42 PE), anti-CD73 (46-0739-42 PerCP), anti-CD90 (12-0909-42 PE), and anti-human leukocyte antigen (HLA)-DR (25-9952-42 PE Cy7), following the manufacturer’s protocol. Isotype controls were used for comparison, and 30,000 events were acquired for each sample using FACSDiva software (Becton Dickinson, San Jose, CA, USA), and the data were analyzed using FlowJo software (TreeStar, Ashland, OR, USA). ASCs were also analyzed for their in vitro differentiation potential into adipogenic (A10070-01), chondrogenic (A10071-01), and osteogenic (A10072-01) lineages using specific differentiation kits (StemPro from Gibco, New York, NY, USA) in 6-well plates according to the manufacturer’s protocol. Differentiations were confirmed using Oil Red O (adipogenic), Alcian Blue (chondrogenic), and Alizarin Red S (osteogenic) dyes. Images of the differentiated cells were captured using a camera attached to an inverted light optical microscope (Zeiss AxioVert, Oberkochen, Germany) with Zeiss Imaging Software. Control differentiation experiments were performed and stained under identical conditions, except for the use of a maintenance culture medium.

### 2.2. Ovarian Carcinoma Cell Lines

The two ovarian adenocarcinoma cell lines, OVCAR3 and SKOV3, were purchased from the Rio de Janeiro Cell Bank (BCRJ) and cultured in DMEM/Ham’s F-12 medium supplemented with 10% FBS and 1% AA. The cultures were maintained in a standard incubator at 37 °C and 5% CO_2_. These cells were chosen because of their distinct characteristics (origin, phenotype, growth, and invasion profile), making the two model lines complementary, allowing the investigation of different aspects of ovarian cancer biology and the identification of specific therapeutic strategies for each tumor profile.

### 2.3. Conditioned Medium Collection

Conditioned medium (CM) from ASCs, OVCAR3, and SKOV3 cell lines was obtained from cultures at 80% confluence in 175 cm^2^ T-flasks (±2.0 × 10^6^ cells/flask). The culture medium was removed, and the adhered cells were washed twice with phosphate-buffered saline (PBS). Subsequently, 25 mL of fresh DMEM/F12 medium, devoid of FBS and AA, was added to the cultures for 48 h. The CM was collected and centrifuged at 300× *g* for 5 min, filtered through a 0.22 μm filter (Millipore, Billerica, MA, USA), and aliquoted for storage at −20 °C until further use.

### 2.4. Isolation of Small Extracellular Vesicles

A portion of the collected CM was processed for the isolation of small extracellular vesicles (sEVs) to evaluate their impact on tumor progression, as described below. Following collection, the CM of ASCs and ovarian cancer (OC) cells was subjected to differential centrifugation at 300× *g* for 10 min and 2000× *g* for 20 min to remove cellular debris. The supernatant was then filtered through a 0.22 μm membrane and cryopreserved at −80 °C for subsequent analysis. Filtered CM was thawed and concentrated using Amicon^®^ Ultra 10 kDa centrifugal filter units (UFC901024; Sigma-Aldrich/Merck, Darmstadt, Germany) by centrifuging 15 mL of CM per tube until the final volume of 500 μL was achieved. The concentrated CM was then subjected to size exclusion chromatography (SEC) using qEVoriginal^®^ Gen 2/35 nm columns (ICO-35; Izon Science, Medford, OR, USA) in accordance with the manufacturer’s protocol. In brief, 500 μL of concentrated CM was loaded onto the top of the column, followed by the addition and subsequent disposal of 2.5 mL of sterile filtered PBS to remove non-vesicular components. The sEVs-enriched fraction was then collected after adding 2 mL of PBS to the column. This fraction was further concentrated to a final volume of 250 μL using Amicon^®^ Ultra 3 kDa centrifugal filter units (UFC800324). Finally, sEVs-containing samples were identified and stored at −80 °C until further use.

### 2.5. Characterization of sEVs by Nanoparticle Tracking Analyses

To determine the size distribution and concentration of isolated sEVs, nanoparticle tracking analyses (NTA) were performed. A 10 μL aliquot of concentrated sEVs solution from each sample was diluted in 990 μL of sterile filtered PBS (1:100) to analyze particles ranging 10 to 1000 nm in diameter using a NanoSight NS500 (Malvern Panalytical, Malvern, UK), following the manufacturer’s instructions. For all measurements, camera settings were standardized with a camera level of 15, and three 30 s videos were recorded for each sample. The resulting videos were analyzed using Nano Sight NTA 3.2 software version 3.2.16, with the detection threshold set to 5.

### 2.6. Transmission Electron Microscopy

The ultrastructural morphological characterization of sEVs was performed using transmission electron microscopy (TEM). Briefly, sEVs samples were fixed with 2% paraformaldehyde, and 5 μL of each sample was applied to Formvar-carbon-coated electron microscopy grids (CF200-NI-50; Electron Microscopy Sciences, Hatfield, PA, USA), allowing adsorption for 20 min at room temperature (RT). The grids were then washed with 100 μL of PBS, transferred to 50 μL of 1% glutaraldehyde solution, and incubated for 5 min, followed by three washes with PBS. Finally, the grids were stained with 10 μL of UAR-EMS Uranyl Acetate replacement dye for 15 min. The grids were then carefully removed using stainless steel handles, excess fluid was blotted with filter paper, and after drying, the prepared sEVs were visualized using a transmission electron microscope.

### 2.7. Micro BCA Assay

The total protein concentration in each sEV sample was quantified using the Micro BCA^TM^ Protein Assay colorimetric kit (23235; Thermo Scientific^TM^, Rockford, IL, USA) according to the manufacturer’s protocol. Prior to measurement, sEVs were lysed at a 1:4 *v*/*v* ratio with cell extraction buffer (FNN0011, Invitrogen-Thermo Scientific^TM^) supplemented with 1 mM phenylmethylsulphonyl fluoride (PMSF) (36978; Thermo Scientific^TM^) and 5% protease inhibitor (PI) cocktail (P-2714; Sigma-Aldrich/Merck) for 30 min on ice, with vortexing at 10 min intervals. The lysate was then centrifuged at 13,000 rpm for 10 min at 4 °C, and the supernatant was mixed with the Micro BCA working reagent. The mixture was transferred in duplicate to 96-well plates (650161; Greiner Bio-One, Kremsmünster, Austria) sealed with sealing tapes and incubated at 37 °C for 2 h. At the end, the absorbance was measured at 562 nm using a microplate reader. A bovine serum albumin (BSA)-based standard curve was simultaneously prepared to calculate the protein concentration in μg/mL for each sample.

### 2.8. Cellular Uptake and Internalization of sEVs

To assess the uptake of sEVs across cell lines, sEVs (20 μg/sample) were labeled with 7.5 μM stock solution of Vybrant^®^ CFDA SE Cell Tracker Kit (V12883; Invitrogen-Thermo Scientific^TM^) for 15 min at RT, protecting from light. After labeling, the samples were washed with sterile filtered PBS and concentrated using Amicon^®^ Ultra 3 kDa centrifugal filter units. The PBS solution containing carboxyfluorescein diacetate succinimidyl ester (often called CFSE)-stained sEVs derived from ASCs or OC cell lines was diluted culture medium to a final concentration of 20 μg/mL. The CFSE-stained sEVs were then applied to cells (2.0 × 10^4^ cells per well) cultured in an 8-Chamber Slide System (177380; Nunc™ Lab-Tek™, Thermo Scientific^TM^) pre-treated for cell adhesion. After 24 h of treatment under standard incubator conditions, the culture medium was discarded, and the cells were subjected to the confocal microscopy protocol as described in Section 2.13. Additionally, cells were incubated with the primary antibody anti-GAPDH (1:200; ab9485, Abcam, Cambridge, MA, USA) and the secondary antibody Alexa Fluor 594-conjugated goat anti-rabbit IgG (1:400; #A-11037, Invitrogen-Thermo Scientific^TM^) to highlight cell morphology. The Vectashield^®^ Mounting Medium with DAPI (H-1200; Vector Laboratories, Newark, NJ, USA) was also used to label the nucleus. The internalization of CFSE-stained sEVs (excitation at 492 nm and emission at 517 nm) by the cells was confirmed and visualized using confocal microscopy (Leica Microsystems, Wetzlar, Germany) at 63× magnification. Image acquisition was performed using Leica X software (Leica Microsystems) with the 3D tool extension. Negative controls followed the same protocol, except for the use of PBS prepared with 7.5 µM CFSE, without sEVs.

### 2.9. Colony Formation Assay

The colony formation efficiency of OC cells in different conditions was evaluated by seeding 1.0 × 10^3^ cells per well (3.66 cm^2^) in 12-well plates, which were maintained under standard incubator conditions. Each triplicate was treated with culture medium supplemented with 10% FBS and 1% AA, either without sEVs derived from ASCs (control) or containing 40 µg/mL of ASC-derived sEVs. The culture was maintained for 8 days, with the treatment medium renewed every two days. At the end of the culture period, cells were fixed with methanol for 10 min, stained with 0.2% crystal violet for 30 min, and washed twice with PBS. After drying, each well was photographed, and colonies were counted using Image J software version 1.54f. Colony formation efficiency was calculated using the following formula [24]:

$$Colony\;forming\;efficiency=\left(\frac{Number\;of\;colonies}{Number\;of\;cells\;seeded}\right)\times 100$$

### 2.10. Cell Proliferation Assay

The indirect proliferation capacity of the cells was evaluated by seeding 1.0 × 10^3^ cells per well (0.33 cm^2^) in triplicate in 96-well plates for 6 days. The control medium consists of DMEM/F12 with 10% FBS and 1% AA, while experimental conditions were supplemented with 10 or 40 µg/mL ASC-derived sEVs for OC cells, or 10 and 40 µg/mL of OVCAR3 sEVs, or SKOV3 sEVs for ASCs. The treatment medium was renewed every two days. Every 24 h, the treatment from one triplicate was removed, and a solution containing 5 mg/mL MTT (3-(4,5-dimethylthiazol-2-yl)-2,5-diphenyltetrazolium bromide) in control medium was added to each well for 2 h of incubation at 37 °C and 5% CO_2_. Finally, the formazan crystals were dissolved by adding 100 μL of dimethyl sulfoxide (DMSO), followed by homogenization and absorbance measurement at 550 nm using a microplate reader. Optical density (OD) values were normalized to the readings obtained at the end of the 1st day of culture for each experimental group.

### 2.11. Western Blot Analysis

To assess the effects of secretome on ASCs and OC cell lines, each experimental condition was cultured in triplicate in 75 cm^2^ T-flasks, starting with an initial seeding density of 3.0 × 10^5^ cells. Cultures were maintained for 6 days under control conditions (50% CM from the same cell type) or treated with 50% ASC-derived CM (for OVCAR3 and SKOV3 cells) or 50% CM derived from OVCAR3 and SKOV3 (for ASCs). The treatment medium was mixed with 50% DMEM/F12 containing 10% FBS and 1% AA and was fully replaced on days 0, 2, and 4. At the end of the 6th day, the medium was discarded, and the cells were lysed with RIPA buffer (89901; Thermo Scientific^TM^) supplemented with 1% PI cocktail (P-8340; Sigma-Aldrich/Merck) and maintained for 48 h at 20° C. After thawing on ice, adherent cells were detached using a cell scraper, and the resulting solution was transferred to a tube and maintained on ice for 40 min. The cell homogenate was then centrifuged at 14,000 rpm for 20 min at 4 °C. The supernatant was collected and stored in a biofreezer at 80 °C until protein quantification was performed using the Bradford assay.

Subsequently, 40 µg of protein were stabilized with 1× Laemmli buffer, denatured by heating at 95 °C for 5 min in a dry bath, and loaded onto 10% polyacrylamide gel for SDS-PAGE. Electrophoresis was conducted at 110 V, followed by electrotransfer of proteins to nitrocellulose membranes at 350 mA. The membranes were blocked with 3% non-fat milk powder in a blocking buffer and incubated with the primary antibodies: anti-TGFβII (2 µg/mL; ab308854), anti-ERK1+2 (1:200; ab214362), anti-Snail+Slug (1 µg/mL; ab85936), anti-Smad2+3 (1:200; ab217553), anti-MMP-9 (1:1000; ab76003), anti-E-cadherin (1 µg/mL; ab231303), anti-β Catenin (1:5000; ab32572), anti-Cytokeratin 5 (1:500; ab53121) (Abcam), and anti-erbB2/HER-2 (1:1000; 06-562, Merck/Millipore), diluted in 1% BSA overnight at 4 °C. The membrane was subsequently washed with TBS-T and incubated with a secondary antibody diluted in 1% BSA conjugated to horseradish peroxidase (HRP) (1:5000; ab6721 and ab6789; Abcam) for 1 h and 30 min. Finally, the reaction was produced by adding the chemiluminescent substrate ECL^®^ Selected Western Blotting Detection Reagent System (RPN2236; GE Healthcare, Uppsala, Sweden), and the intensity of each band quantified using the optical densitometry index (IOD) normalized by the intensity of the endogenous marker β-actin (1:200; sc-47778). Data analyses were performed using the Image J software version 1.54f (National Institutes of Health, Bethesda, MD, USA).

sEVs were also characterized by Western blotting using established sEV markers. A total of 10 µg per sample (pool) was mixed with lysis buffer (FNN0011) supplemented with 1 mM PMSF (36978) and 5% PI cocktail (P-2714) in a 1:1 *v*/*v* ratio. The samples were incubated on ice for 1 h with intermittent stirring. Following this, the samples were processed according to the standard Western blot protocol and incubated with primary antibodies: anti-CD63 (1:1000; ab193349) and anti-CD81 (2 µg/mL; ab79559) (Abcam). Reactivity was confirmed as previously described.

### 2.12. Indirect Co-Culture Assay

To evaluate the effects of the interaction between OVCAR3 and SKOV3 cells with ASCs in the same in vitro environment, an indirect co-culture assay was performed. Transwell inserts for 12-well plates (665610; Greiner Bio-One, Kremsmünster, Austria) with a polycarbonate membrane of 0.4 μm pore size, allowing communication between cell lines via the release of EVs, were used. Cells were seeded at a density of 2.0 × 10^4^ above the microporous membrane or at 1.0 × 10^4^ in the wells containing a 13 mm diameter round coverslip. Each culture was maintained in DMEM/F12 medium with 10% FBS and 1% AA for 6 days, with partial medium renewal (50%) every 48 h, allowing the cells to maintain the conditioned medium as treatment. At the end of the culture period, the cells adhered to the coverslip surface were analyzed using confocal microscopy. The control group consisted of a monoculture using the same cell line in both compartments, maintained under the same medium renewal protocol.

To allow comparisons between each experimental condition without interference from the confocal microscopy setup, the photomicrographs were initially captured from the control group. Capture parameters, including laser intensity, number of steps, Z-step size, resolution, reading speed, pinhole aperture, and others, were standardized based on the control group and subsequently applied to the respective treatment conditions for cells labeled with the same proteins. This approach ensured that any observed differences in the intensity of labeled proteins were attributed solely to the treatment conditions rather than to variations in the imaging setup.

### 2.13. Confocal Microscopy

At the end of the indirect co-culture assay, the cells on the coverslips were fixed with 4% paraformaldehyde for 20 min, washed three times with PBS, and incubated with PBS-Tween-20 (1:100) (T-PBS) solution for 5 min. Following this, the cells were permeabilized with 0.1% Triton X-100 solution for 10 min and blocked with 5% BSA for 30 min at RT. Next, the cultures were incubated overnight at 4 °C with a combination of primary antibodies: anti-p38alpha (1:200; ab170099), anti-Survivin (5 µg/mL; ab24479), anti-NF-κB (1:200; ab7970), anti-β Catenin (1:100; ab32572) (Abcam), anti-ERK1+2 (1:200; sc-514302), anti-BMP2+4 (1:200; sc-6267), anti-Cytokeratin 5 (1:50; sc-17090), and anti-Wnt-2 (1:200; sc-5208) (Santa Cruz Biotechnology, Dallas, USA), all diluted in 5% BSA. After three washes with T-PBS, the slides were incubated with the appropriate IgG secondary antibody (Alexa Fluor 488, 1:400; ab150077 or Alexa Fluor 594, 1:400; #A-11037 or #A-11058, Invitrogen-Thermo Scientific^TM^) for each primary antibody in 5% BSA for 1 h and 30 min at RT. Following incubation, the coverslips were washed three times with PBS and then mounted with antifade mounting medium conjugated with DAPI (Vectashield^®^ H-1200; Vector Laboratories). The samples were subsequently analyzed under a confocal fluorescence microscope (Leica TCS SP8, Leica Microsystems) using various magnifications and Leica X (Leica Microsystems) and Las X Office (Leica Microsystems) software for image acquisition and analysis.

### 2.14. Invasion Assay

The invasiveness of OVCAR3, SKOV3, and ASCs treated with CM was assessed using 12-well plates containing 8 μm micropore Transwell inserts (665638; Thincert^®^, Greiner Bio-One). Each insert was coated with a thin Geltrex^®^ membrane (200 μL/cm^2^) (A1569601; Gibco, Thermo Scientific^TM^), which mimics the biological basement membrane and occludes the polyethylene terephthalate (PET) micropores. After allowing 30 min for the formation of the Geltrex^®^ matrix, cells were seeded onto the upper compartment (6.0 × 10^4^) without serum, while the lower compartment was treated with CM supplemented with 10% FBS to act as a chemical attractant. Cells were incubated at 37 °C and 5% CO_2_ for 24 h. After this period, the inserts were fixed in methanol for 10 min, stained with 0.2% crystal violet solution for 30 min, and washed once with PBS. Non-migratory cells were removed using a cotton swab, while the cells that migrated to the bottom of the insert were photographed in four fields under an inverted microscope (Zeiss AxioVert^®^) at 10× magnification. Migrated cells were counted using Image J software version 1.54f, and the results were normalized to the respective control (the same CM from the originating cell type) subjected to identical staining and quantification procedures.

### 2.15. Wound Healing Migration Assay

The migratory potential of ASCs and OC cells in response to dose-dependent sEVs treatments (10, 20, and 40 µg/mL or PBS as control) was assessed in triplicate in 96-well plates containing DMEM/F12 medium supplemented with 2% FBS. Cells (1.0 × 10^4^) were seeded and allowed to form a monolayer over 24 h under standard conditions. A scratch wound was then created in each well using a 200 µL tip. After washing with PBS to remove cellular debris, the respective treatments were added, and an initial photomicrograph was taken using an inverted microscope (Zeiss AxioVert^®^) at 10× magnification. After 24 h of culture, the same area was photographed again and compared with the initial images (0 h) to assess the percentage of wound closure. The wound closure was quantified by measuring the difference in the scratched area before and after 24 h of treatment.

### 2.16. Extracellular Signaling Analysis by Multiplex Assay

Quantitative measurements of extracellular signaling molecules secreted by ASCs and OC cells in the CM secretome were conducted using the ProcartaPlex™ magnetic bead-based multiplex immunoassay (Thermo Scientific™) according to the manufacturer’s instructions. This approach allowed the simultaneous measurement of multiple factors involved in tumor progression, immune modulation, and extracellular matrix remodeling. To achieve this, the CM from ASCs and OC cells was collected and processed as previously described, followed by concentration using Amicon^®^ Ultra 10 kDa centrifugal filter units (UFC901024; Sigma-Aldrich/Merck) to a final volume of 500 μL. The total protein concentration in each sample was quantified using the Micro BCA^TM^ Protein Assay colorimetric kit (23235; Thermo Scientific™) as outlined before. The protein concentrations of interleukin-15 (IL-15), vascular endothelial growth factor A (VEGF-A), tumor necrosis factor alpha (TNF-α), TNF-related apoptosis-inducing ligand (TRAIL), Tenascin-C, tissue inhibitor of metalloproteinases-1 (TIMP-1), matrix metalloproteinase 2 (MMP-2), and stromal-derived factor 1 alpha (SDF-1α) were determined by measuring the fluorescence levels detected using a MAGPIX^®^ System (Luminex® Corporation, Austin, TX, USA). Each sample was analyzed in three technical and biological replicates, and the results were normalized to the total protein content, expressed as pg/μg.

### 2.17. Protein–Protein Interaction (PPI) and Overall Survival of OC Patients

After identifying the molecular targets altered by the treatments, we analyzed the associated biological processes using the STRING 11.0 database (https://cn.string-db.org/) (accessed on 23 February 2025) to construct a protein–protein interaction (PPI) network. Subsequently, PANTHER classification (http://www.pantherdb.org/) (accessed on 23 February 2025) was employed to perform enrichment analysis based on molecular function and protein class. Building upon this molecular network influenced by extracellular mediators, we utilized GEPIA2 (http://gepia2.cancer-pku.cn/#survival) (accessed on 23 February 2025) [30] to investigate gene expression profiles and assess the association between high- and low-risk groups and overall survival in patients based on the TCGA dataset for ovarian serous cystadenocarcinoma (OV). To further stratify patients, we evaluated correlations through Cox regression analysis of overall survival.

### 2.18. Statistical Analysis

The statistical analysis was performed using analysis of variance (one-way ANOVA) with independent factors, complemented with Tukey’s test for multiple comparisons. For nonparametric data, the Kruskal–Wallis test was applied, complemented by the Dunn test. When comparing two groups, the unpaired t-test was used. All results were expressed as mean ± standard error of the mean (SEM) and analyzed with the GraphPad Prism 9.0 software (GraphPad Software, Inc.; Boston, MA, USA). Graphs were presented considering a significance level of 5% (*p* < 0.05). All experiments were conducted in at least three technical and biological replicates.

## 3. Results

### 3.1. EVs from ASCs and OC Cell Lines Were Characterized as sEVs and Effectively Internalized During Treatment

**Figure 1:**
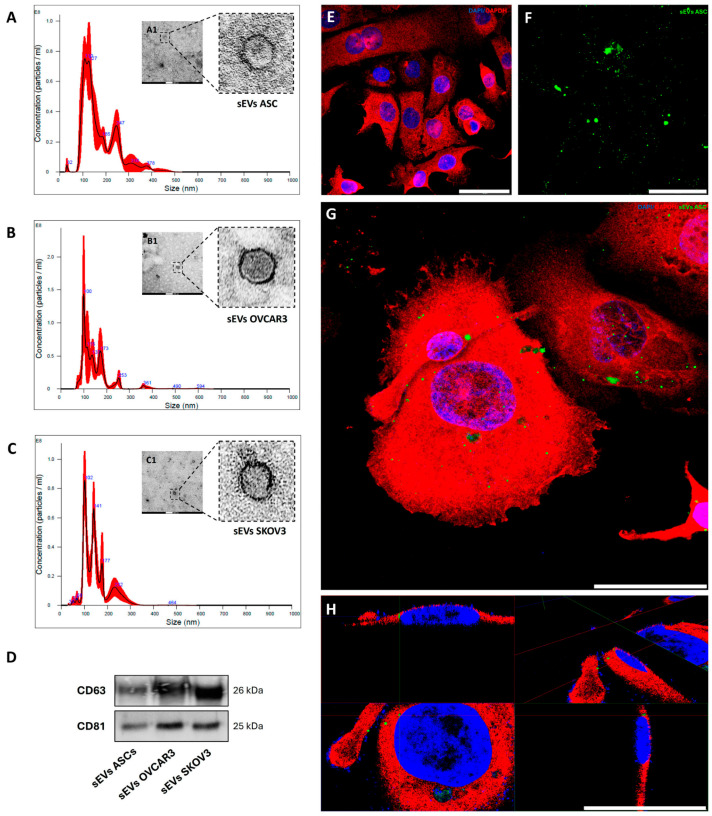
Characterization and internalization of sEVs. (**A**–**C**) Representative images of NTA performed with sEVs from ASCs (**A**), OVCAR3 (**B**), and SKOV3 (**C**) cells. (**A1**–**C1**) Representative TEM images of sEVs from ASCs (**A1**), OVCAR3 (**B1**), and SKOV3 (**C1**) cells. (**D**) Western blotting analysis of EVs surface markers CD63 and CD81 from a pool of samples from each cell line. (**E**) Negative control. Representative image of cells treated with CFSE solution without sEVs. (**F**) CFSE-sEVs on a cell-free adhesion slide. (**G**) Merged image of cells treated for 24 h with 20 µg/mL CFSE-sEVs. Endogenous marker GAPDH (red), nucleus (blue), CFSE-sEVs (green). (**H**) Representative image of the 3D reconstruction of image G highlighting the internalization of CFSE-sEVs into the cytoplasm. Scale bars = 100 nm (**A1**,**B1**), 200 nm (**C1**), and 50 µm (**E**–**H**).

The EVs obtained from the CM of each cell line and isolated by the SEC method were characterized with NTA (Figure 1A–C), which shows a mean size of 188.5 nm ± 18.4 for ASC-sEVs, 160.4 nm ± 18.5 for OVCAR3-sEVs, and 197.6 nm ± 24.5 for SKOV3-sEVs; this size range, which was similar among different EVs, characterized them as small EVs (sEVs). All samples also show peaks above 200 nm, possibly related to some sEV aggregates. Additionally, NTA revealed a particle concentration of 5.81 × 10^9^ ± 9.76 × 10^8^ ASC-sEVs/mL, 5.18 × 10^9^ ± 2.02 × 10^9^ OVCAR3-sEVs/mL, and 5.26 × 10^9^ ± 7.54 × 10^8^ SKOV3-sEVs/mL. Regarding Micro BCA assay, ASC-sEVs presented a mean protein concentration of 274.6 µg/mL ± 8.7, while for sEVs from OC cells, the mean was 308.4 µg/mL ± 9.4 (OVCAR3-sEVs) and 288.5 µg/mL ± 5.3 (SKOV3-sEVs). No significant differences were observed in the analyses of size, number of particles, and protein concentration of sEVs.

The sEVs were ultramorphologically characterized by TEM. As expected, TEM images show sEVs in the 50/200 nm range size measured by NTA (Figure 1(A1–C1)). Some sEVs also appeared in aggregates, with vesicles of varying sizes observed, potentially due to fixation artifacts. sEVs were also characterized by positive reactions with EV surface markers CD63 and CD81 (Figure 1D).

Cells analyzed by confocal microscopy confirmed cellular uptake and internalization of CFSE-sEVs (Figure 1E–H). sEVs stained at a concentration of 20 µg/mL appear widely distributed throughout cells after 24 h of treatment. The use of the endogenous GAPDH protein labeled in red highlights the CFSE-sEVs in green taken up intracellularly by the cells (Figure 1G). The sEVs were located mainly in the cytoplasm and cell periphery, but some were also located in the nucleus. This was only observed using a confocal microscope and 3D reconstruction videos from z-stacks (Figure 1H). No differences regarding the uptake and internalization of sEVs were apparent between the cell lines.

### 3.2. ASC-Derived sEVs Enhance the Colony-Forming Efficiency, Whereas ASC Secretome Upregulates the Expression of Proteins Associated with the TGFβ Signaling Pathway

**Figure 2:**
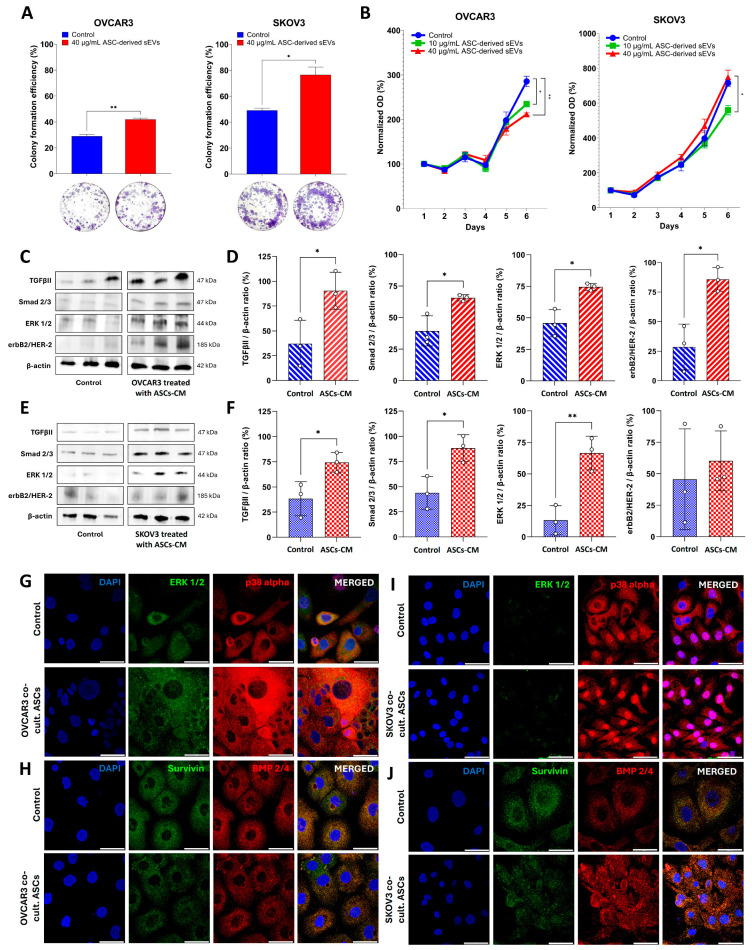
Effects of ASC-derived sEVs or CM secretome on OC colony formation efficiency (**A**), proliferation rate (**B**), and protein expression of TGFβ signaling pathway members assessed by Western blot (**C**–**F**) and confocal microscopy (**G**–**J**). (**A**) Colony formation efficiency was calculated as the ratio of the number of colonies formed to the number of cells seeded. Representative images show OC colonies after 8 days of culture and after staining with 0.2% crystal violet. (**B**) Proliferation rate of the OC groups normalized by the densitometric quantification of each group on day 1 of culture. (**C**,**E**) Western blotting bands showing the protein levels quantified for TGFβII, Smad 2/3, ERK 1/2, and erbB2/HER-2 in OVCAR3 (**D**) and SKOV3 (**F**) cells after treatment of triplicates with 50% ASC-derived CM secretome for 6 days and normalization by β-actin. Representative images of confocal microscopy analysis showing the expression of ERK1/2, p38alpha, Survivin, and BMP2/4 marked in OVCAR3 (**G**,**H**) and SKOV3 (**I**,**J**) cells after 6 days of culture under control conditions (monoculture) and indirectly co-cultured with ASCs. Statistical significance is indicated as * *p* < 0.05 and ** *p* < 0.01. Scale bars = 50 µm.

The role of ASCs in tumor development remains controversial [7,31,32]. To address this, we investigated the effects of extracellular signaling molecules secreted by ASCs, either through their CM secretome or via sEVs, on colony-forming efficiency, cell proliferation, and tumor-related protein expression in OC-derived cell lines. Our findings demonstrated that treatment with 40 µg/mL of ASC-derived sEVs increased (*p* < 0.01) the colony formation efficiency of both OC cell lines after 8 days of culture (Figure 2A). Despite that, when cultured for 6 days, a decrease (*p* < 0.05) in the proliferation rate was observed in OVCAR3 cells treated with 10 or 40 µg/mL of ASC-derived sEVs compared to the control group (Figure 2B). In contrast, no differences in SKOV3 proliferation were observed between the control and ASC-derived sEVs treatments. Notably, the higher dosage of ASC-derived sEVs (40 µg/mL) induced a greater (*p* < 0.05) proliferation rate in SKOV3 cells compared to the lower dosage (10 µg/mL) (Figure 2B). These findings suggest a distinct effect of sEVs on OC cell proliferation, which may be concentration-dependent and cell-line-specific.

Simultaneously, OC cell lines treated with ASC-derived CM secretome for 6 days were analyzed by Western blotting to quantify the intensity of each band for the expression of functional factors associated with tumor growth and progression (Figure 2C–F). Following treatment with 50% ASC-derived CM, both OC cell lines exhibited an increase (*p* < 0.05) in the expression of supportive cytokines such as TGFβII and its signaling mediator, Smad 2/3 (Figure 2D–F). Similarly, protein kinases from the MAPK family, including ERK1 (MAPK3) and ERK2 (MAPK1), showed elevated expression levels (*p* < 0.05), along with the enhancer of kinase-mediated activation erbB2/HER-2 (*p* < 0.05) (Figure 2D–F). Notably, despite the SKOV3 cells demonstrated an increase in erbB2/HER-2 expression, the change was not statistically significant (Figure 2F).

**Figure 3:**
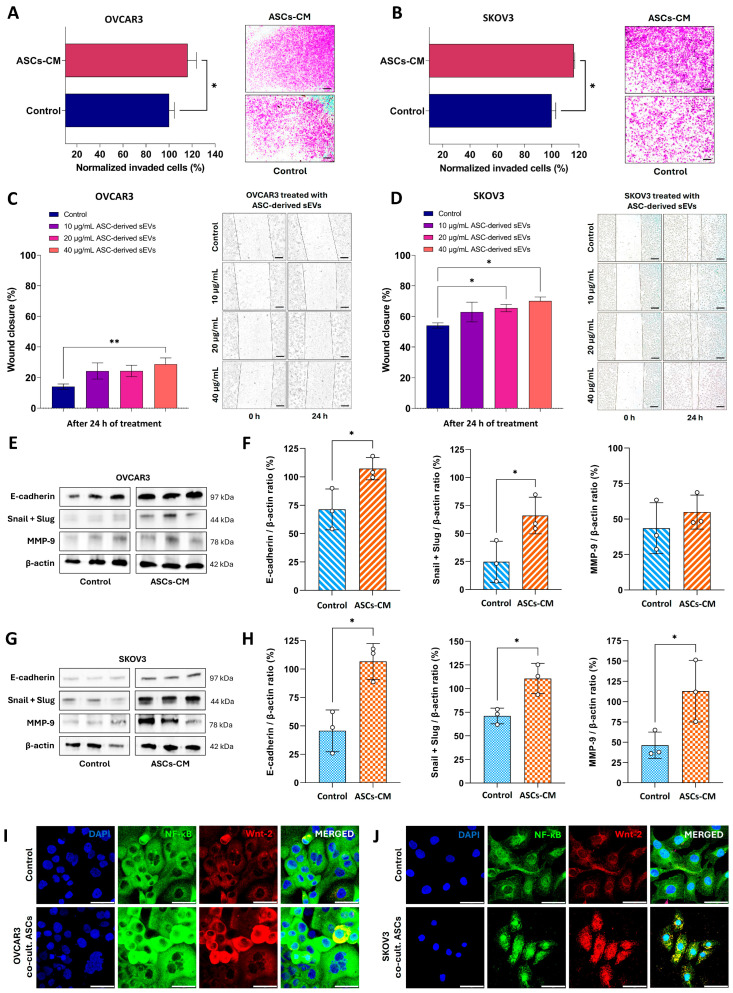
Analysis of the impact of ASC-derived extracellular signaling molecules from CM and sEVs on the invasive and migratory capacity of OC cells. (**A**,**B**) The invasiveness of OC cells was evaluated using a Transwell assay after 24 h of treatment with 50% of ASC-derived CM. The number of invaded cells was normalized to the respective control. Representative images of OVCAR3 (**A**) and SKOV3 (**B**) cells that trespass the Geltrex^®^ membrane. (**C**,**D**) The migratory capacity of OC cells was assessed via wound healing assay. Representative images depict the wound closure in the monolayer of OVCAR3 (**C**) and SKOV3 (**D**) cells at 0 h and after 24 h of treatment with increasing doses of ASC-derived sEVs. Western blotting bands obtained from the triplicates of OVCAR3 (**E**) and SKOV3 (**G**) cells treated for 6 days with 50% ASC-derived CM secretome. Quantitative analysis of E-cadherin, Snail+Slug, and MMP-9 protein levels of OVCAR3 (**F**) and SKOV3 (**H**) cells normalized to β-actin. Representative confocal microscopy images illustrating the expression of NF-κB and Wnt-2 in OVCAR3 (**I**) and SKOV3 (**J**) cells under control conditions or after 6 days of indirect co-cultured with ASCs. Statistical significance is indicated as * *p* < 0.05 and ** *p* < 0.01. Scale bars = 50 µm (white), 200 μm (black).

These results led us to analyze the expression of certain tumor-associated proteins by confocal microscopy following 6 days of indirect co-culture between OC cell lines and ASCs in a non-contact cell-to-cell communication. Qualitative observations revealed an overall increase in the cellular expression of tumor-supportive markers in OC cells co-cultured with ASCs (Figure 2G,I,J). Specifically, the expression of ERK 1/2 and p38 alpha (MAPK14), members of the MAP kinase family, was notably elevated in the cytoplasm of OVCAR3 cells and in the nucleus and cytoplasm of SKOV3 cells (Figure 2G and Figure 3I, respectively). A similar increase was observed for BMP 2/4, a component of the TGFβ superfamily, particularly in SKOV3 cells (Figure 2J). However, no significative differences were observed in the expression of Survivin, a protein encoded by the inhibitor of the apoptosis gene family, in either OC cell line (Figure 2H,J) or in BMP 2/4 expression in OVCAR3 cells (Figure 2H). These results indicate that the communication between ASCs and OC cells significantly modulates the expression of various tumor-promoting proteins, including key members of the MAPK pathway (ERK 1/2 and p38 alpha) and the TGFβ superfamily (BMP 2/4). Our findings highlight the influence of ASCs on the molecular landscape of OC cells, potentially enhancing their metastatic and tumor-supportive capabilities.

### 3.3. ASC-Derived Extracellular Signaling Molecules Increase the Invasive and Migratory Capacity of OC Cell Lines, Promoting a Hybrid State Epithelial/Mesenchymal Phenotype

Given the evidence that ASCs release bioactive molecules associated with tumor growth, we sought to determine whether these extracellular mediators alter the metastatic potential of OC cells by assessing their invasive and migratory capacities. As illustrated in Figure 3A,B, treatment with ASC-derived CM secretome enhanced (*p* < 0.05) the invasiveness of both OC cells through the Geltrex^®^ membrane. Subsequently, the migratory potential of OC cells was evaluated using a wound healing assay with ASC-derived sEVs as treatment. As expected, both OC cell lines migrated into the scratched area, reducing the wound’s free surface over the 24 h observation period (Figure 3C,D). This effect was dose-dependent, with significant wound closure observed at the 40 µg/mL dosage of ASC-derived sEVs for OVCAR3 cells (28.8%, *p* < 0.01) (Figure 3C). For SKOV3 cells, significant closure occurred at both 20 µg/mL (65.5%, *p* < 0.05) and 40 µg/mL (70.1%, *p* < 0.05) dosages of ASC-derived sEVs (Figure 3D). These results suggested that some contents secreted by ASCs may play a role in promoting tumor cell migration and dissemination.

In this context, we quantified by densitometric analysis proteins known to be associated with invasion, migration, and metastasis (Figure 3E–H). E-cadherin, the calcium-dependent cell–cell adhesion glycoprotein, was significantly increased (*p* < 0.05) in OC cells after 6 days of culture with 50% ASC-derived CM secretome, along with other proteins involved in tissue remodeling, such as Snail/Slug and MMP-9 (Figure 3E–H). The latter was one significantly increased in SKOV3 cells (Figure 3G,H).

The simultaneous expression of E-cadherin, an epithelial marker, and Snail/Slug, a mesenchymal marker, in OC cells, suggests the promotion of a hybrid epithelial/mesenchymal phenotype by ASC secretome. Upon analyses of confocal images, we observed an increase in the expression of signaling protein Wnt-2, predominantly in the cytoplasm of OC cells, as well as an upregulation of the transcriptional regulator NF-κB in both the cytoplasm and nucleus of OC cells following indirect co-culture with ASCs (Figure 3I,J). Taken together, these data highlight the potential function of ASCs to influence the invasive and metastatic behavior of OC cells, further underscoring the complex role of ASCs in tumor progression.

### 3.4. Extracellular Signaling Molecules from OC Cell Lines Change the Phenotypic Characteristics of ASCs to Acquire Functional Properties That Support Tumor Growth

**Figure 4:**
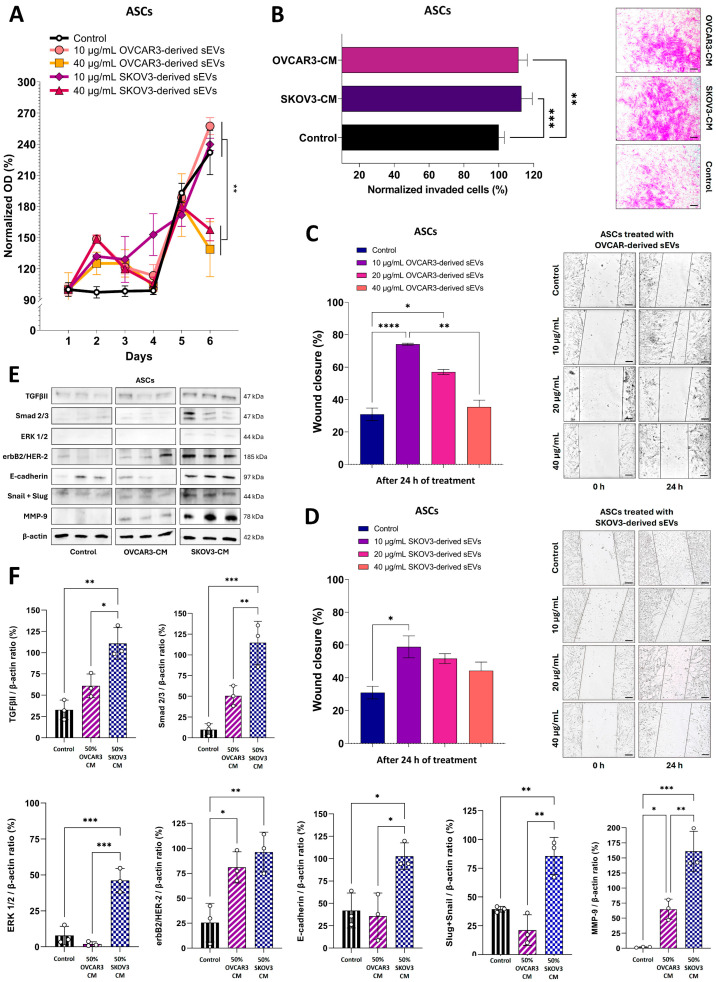
Impact of extracellular signaling molecules from OC-derived CM or their sEVs on the proliferation, invasiveness, and tumor-associated protein expression in ASCs. (**A**) The proliferation rate of ASCs was assessed using the MTT assay after 6 days of culture with two concentrations (10 and 40 µg/mL) of sEVs derived from OVCAR3 or SKOV3 cells. The control group received vehicle treatment (PBS). (**B**) The invasive potential of ASCs was evaluated using the Transwell assay following 24 h of treatment with 50% CM from either OVCAR3 or SKOV3 cells. Invaded cells were normalized to the respective control. Representative images show ASCs that crossed the Geltrex^®^ membrane under each treatment condition. (**C**,**D**) The migratory capacity of ASCs was assessed using the wound healing assay. Representative images show the wound area in ASC monolayer treated with increasing doses of OVCAR3-derived sEVs (**C**) and SKOV3-derived sEVs (**D**) at the initial point (0 h) and after 24 h of treatment. (**E**) Western blotting was performed on ASCs (**E**) treated for 6 days with 50% OVCAR3 or SKOV3 CM. Bands represent the three technical and biological replicates for each treatment. (**F**) Quantitative analysis of TGFβII, Smad 2+3, ERK 1+2, erbB2/HER-2, E-cadherin, Snail+Slug, and MMP-9 protein levels of ASCs normalized by β-actin. Statistical significance is indicated as * *p* < 0.05, ** *p* < 0.01, *** *p* < 0.001, and **** *p* < 0.0001. Scale bars = 200 µm.

To elucidate the impact of bidirectional communication between OC cells and ASCs, which were successfully characterized as MSCs (Appendix A), we investigated how extracellular signaling molecules released from the tumor cells may influence some characteristics of ASCs. As demonstrated in Figure 4A, treatment with sEVs from both OC cell lines caused a dose-dependent effect in the proliferation rate of ASCs after 6 days of culture. While ASCs treated with 10 µg/mL of sEVs from both OVCAR3 and SKOV3 cells showed proliferation levels similar to the control, a higher dosage of 40 µg/mL from OC cells significantly reduced (*p* < 0.05) ASC proliferation (Figure 4A). The treatments with OC-derived sEVs also differed (*p* < 0.05) between the two dosages (Figure 4A). Subsequently, ASCs were treated with 50% OC-derived CM to assess the impact on their invasive potential. Treatment with CM secretome derived from either OVCAR3 or SKOV3 cells resulted in an increase in the invasiveness of ASCs (*p* < 0.01) (Figure 4B). These findings were further supported when we evaluated the effects of OC-derived sEVs on ASC migration using the wound healing assay (Figure 4C,D). Notably, the migratory capacity of ASCs significantly increased following treatment with 10 µg/mL of sEVs from OVCAR3 (74.1%) or SKOV3-derived sEVs (58.9%) compared to control after 24 h of culture. However, higher doses of sEVs resulted in a reduced increase in the migration of ASCs, with no significant changes observed, except for treatment with 20 µg/mL of OVCAR3-derived sEVs, which achieved 57.1% of wound closure (*p* < 0.05).

Recognizing that a 24 h treatment with CM secretome or sEVs derived from OC cell lines modulates cellular trafficking of ASCs, thereby influencing the tissue microenvironment, we further explored the expression of proteins associated with cell proliferation, migration, and metastatic potential through Western blot analysis. The results revealed a pronounced susceptibility of ASCs to the influence of OC-derived CM, particularly from SKOV3 cells (Figure 4E,F).

After 6 days of treatment with 50% SKOV3-derived CM secretome, an increase (*p* < 0.01) was observed in the levels of TGβII receptor and its downstream transducer Smad2/3 (*p* < 0.001). Additionally, elevated expression levels of ERK1/2 from the MAPK family and the oncogene activator erbB2/HER-2 (*p* < 0.01) were detected, with the latter also showing an increase (*p* < 0.05) following treatment with OVCAR3-derived CM (Figure 4E,F). Collectively, these findings underscore the ability of extracellular signaling molecules secreted by OC cells to drive the upregulation of tumor-associated proteins in ASCs, further highlighting their influence on the TME.

**Figure 5:**
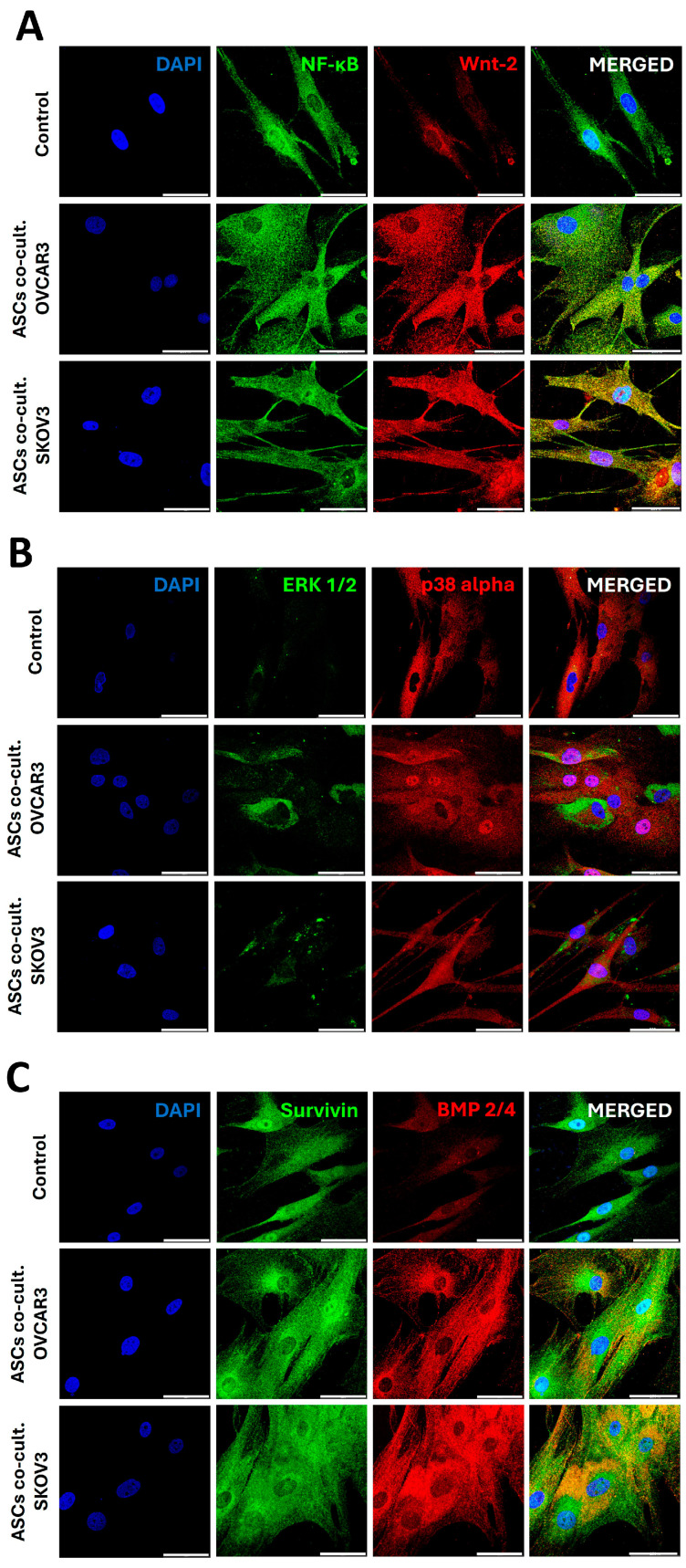
Impact of extracellular signaling molecules secreted by OC cells on ASCs in an indirect co-culture system on tumor-associated proteins. Representative confocal microscopy images of NF-κB and Wnt-2 (**A**), ERK1/2 and p38alpha (**B**), and Survivin and BMP2/4 (**C**) expression in ASCs cultured under control conditions (monoculture) or in indirect co-cultured with OVCAR3 or SKOV3 cells for 6 days. Scale bars = 50 µm.

This phenomenon extended to proteins linked to cellular integrity, such as E-cadherin and MMP-9, which showed elevated levels (*p* < 0.05) following treatment with SKOV3-derived CM, while MMP-9 was increased in ASCs treated with OVCAR3-CM (Figure 4E,F). Snail/Slug levels were also higher in ASCs treated with SKOV3-CM compared to control and OVCAR3-CM (Figure 4E,F). This result, combined with the increased expression of E-cadherin, indicates a transition to a hybrid phenotypic state in ASCs driven by the SKOV3 secretome. Of note, a strong upregulation of Wnt-2 protein was observed in the cytoplasm of ASCs following indirect co-culture with OVCAR3 cells and in the nucleus when co-cultured with SKOV3 cells, compared to the control monoculture (Figure 5A). Interestingly, previous findings demonstrated an elevated expression of Wnt-2 in OC cells indirectly co-cultured with ASCs, suggesting a reciprocal communication between OC cells and ASCs that amplifies the Wnt-2 signaling pathway. The upregulation of ERK1/2 expression was further confirmed morphologically through confocal microscopy analysis, which revealed the localization of this protein at specific cytoplasmic sites or in perinuclear regions of ASCs after a 6-day indirect co-culture with both OC cell lines (Figure 5B). Additionally, other proteins, including Survivin (distributed throughout the cell, including the nucleus) and BMP2/4 (predominantly cytoplasmic), demonstrated a marked increase in expression in ASCs under the same experimental conditions (Figure 5C). Conversely, the expressions of NF-κB and p38alpha remained apparently unchanged (Figure 5A,B).

Overall, the data demonstrate that OC-derived extracellular signaling molecules, such as sEVs or those present in the secretome within the CM, influence the properties and phenotype of ASCs. Through reciprocal communication between the cell types, OC cells promoted the upregulation of multiple tumor-associated proteins in the ASCs to play an active role in supporting OC progression by modulating the TME, ultimately contributing to the invasiveness and metastatic potential of both OC cell lines.

### 3.5. Treatment with ASC- and OC-Derived CM Secretome Change the Levels of EMT Markers

Given the effects of ASC- or OC-derived CM treatment on E-cadherin and Snail/Slug expression, which suggest a hybrid epithelial/mesenchymal phenotype, we further validated this feature by quantifying Cytokeratin 5 and β-catenin as additional markers of epithelial and mesenchymal characteristics, respectively. 

**Figure 6:**
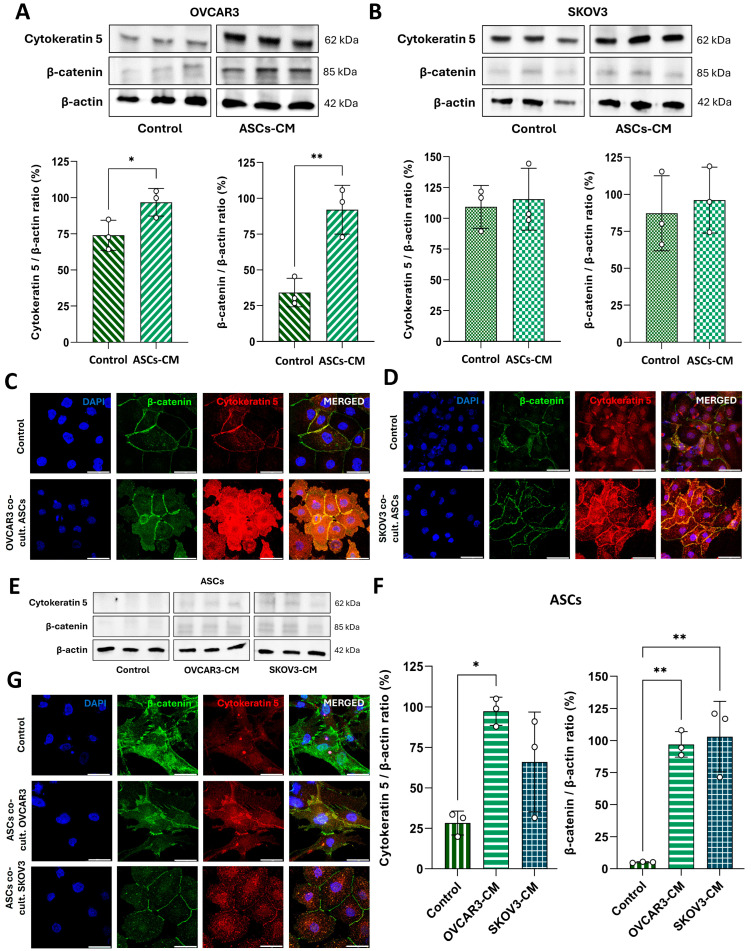
Effects of ASC- or OC-derived CM secretome on protein expression and immunolocalization of epithelial and mesenchymal markers assessed by Western blot. (**A**,**B**,**E**,**F**) Western blotting bands and their quantification showing the protein levels of Cytokeratin 5 and β-catenin in OVCAR3 (**A**), SKOV3 (**B**), and ASCs (**E**,**F**) after treatment with 50% ASC- or OC cell-derived CM secretome for 6 days and normalization by β-actin. Immunolocalization of β-catenin and Cytokeratin 5 in OVCAR3 (**C**), SKOV3 (**D**), and ASCs (**G**) visualized by confocal microscopy after 6 days of indirect co-culture. * *p* < 0.05 and ** *p* < 0.01. Scale bars = 50 µm.

Our findings indicate that OVCAR3 cells exhibited a significant increase (*p* < 0.05) in the expression of both proteins following treatment with 50% ASC-derived CM secretome for 6 days (Figure 6A), whereas SKOV3 cells showed no significant increase (Figure 6B). We also qualitatively evaluated the expression of EMT markers by confocal microscopy after 6 days of indirect co-culture. In this model, an increased expression of Cytokeratin 5 and β-catenin protein was observed in both OC cell lines (Figure 6C,D). In OVCAR3 cells, the expression of both markers was upregulated throughout the cytoplasm (Figure 6C). Conversely, in SKOV3 cells, a pronounced increase was observed only for Cytokeratin 5, while β-catenin expression appeared to decrease in the cytoplasm but was notably enriched at cell junctions (Figure 6D).

In ASCs, treatment with OVCAR3-derived CM showed elevated Cytokeratin 5 expression (*p* < 0.05), while secretome from both OC cells lines significantly upregulated (*p* < 0.01) β-catenin expression (Figure 6E,F). Despite this, immunolocalization analysis revealed a downregulation in β-catenin expression, particularly in cells indirectly co-cultured with SKOV3 (Figure 6G). In contrast, Cytokeratin 5 expression was markedly increased in response to both OC cell lines (Figure 6G).

Together, these results highlight the increased expression of both epithelial and mesenchymal markers, indicating a hybrid phenotypic state, mainly in OC cells, as a result of their interaction with extracellular signaling mediators from ASCs.

### 3.6. Extracellular Signaling Molecules in the CM Secretome Driving the Pro-Tumorigenic State in ASCs and OC Cell Lines

**Figure 7:**
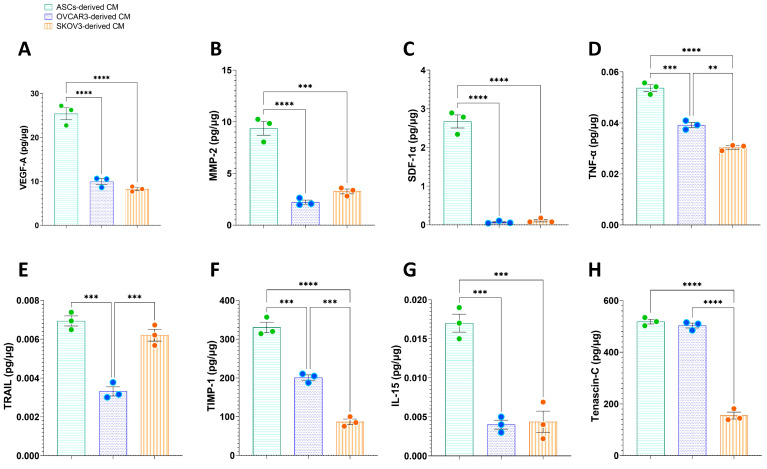
Multiplex analysis of extracellular signaling mediators derived from ASC- and OC-derived CM secretome. The concentrations of VEGF-A (**A**), MMP-2 (**B**), SDF-1α (**C**), TNF-α (**D**), TRAIL (**E**), TIMP-1 (**F**), IL-15 (**G**), and Tenascin-C (**H**) were measured in the CM secretomes of ASCs, OVCAR3, and SKOV3 cell lines under control conditions. Values were obtained in pg/mL and normalized to the total protein concentration of each sample, which results presented as pg/μg of protein. Statistical significance is indicated as ** *p* < 0.01, *** *p* < 0.001, and **** *p* < 0.0001.

Given the influence of ASC- and OC-derived CM in promoting OC progression, we quantified the presence of tumor-associated and tumor-suppressor markers secreted in the CM of ASCs and OC cells under control conditions. Overall, the levels of extracellular signaling molecules were higher in the ASC-derived CM secretome compared to that of OVCAR3 cells and, with few exceptions, also higher than those secreted by SKOV3 cells (Figure 7). Notably, proteins such as VEGF-A, which promotes angiogenesis and increases vascular permeability, MMP-2, involved in tissue remodeling, and SDF-1α, a chemokine crucial for proliferation, migration, and invasion, were significantly elevated (*p* < 0.0001) in the soluble factors derived from ASCs (Figure 7A–C). 

Other cytokines, with a dual role in cancer progression, were also found at higher levels in the ASC-derived secretome compared to OC-CM, including TNF-α (Figure 7D) and TRAIL (Figure 7E). Although both cytokines have pro-apoptotic activity in tumor cells, their effects can shift toward a pro-tumorigenic role depending on the TME context. Similarly, TIMP-1 (Figure 7G) and IL-15 (Figure 7G), which exert anti-tumorigenic functions by inhibiting MMP activity and modulating immune responses, respectively, were significantly elevated (*p* < 0.001) in ASC-derived CM compared to both OC cell lines. Additionally, Tenascin-C, a glycoprotein associated with tumor growth and metastasis, was markedly higher (*p* < 0.0001) in the ASC secretome compared to SKOV3-derived CM (Figure 7H).

When analyzing the extracellular signaling mediators from OC cells, significant differences were observed between the two cell lines. TNF-α, TIMP-1, and Tenascin-C levels were significantly higher (*p* < 0.001) in OVCAR3-derived CM compared to SKOV3-CM (Figure 7D,F,H). Conversely, TRAIL levels were elevated (*p* < 0.01) in the SKOV3-derived secretome compared to OVCAR3-CM (Figure 7E).

These findings highlight the distinct secretory profiles of ASCs and OC cells, emphasizing the complexity of tumor–stroma interactions in the OC microenvironment. Meanwhile, the differences between OVCAR3- and SKOV3-derived CM suggest that OC cells exhibit cell line-specific variations in extracellular signaling. Together, these results reinforce the importance of the cell-derived mediators in influencing cancer progression and suggest that ASCs may play a key role in modulating OC cell behavior through their secretome.

### 3.7. In Silico Functional Enrichment Analysis and Prognosis Assessment of OC Patients Were Influenced Based on Molecular Targets Altered by Bioactive Molecules Secreted by ASCs and OC Cells

**Figure 8:**
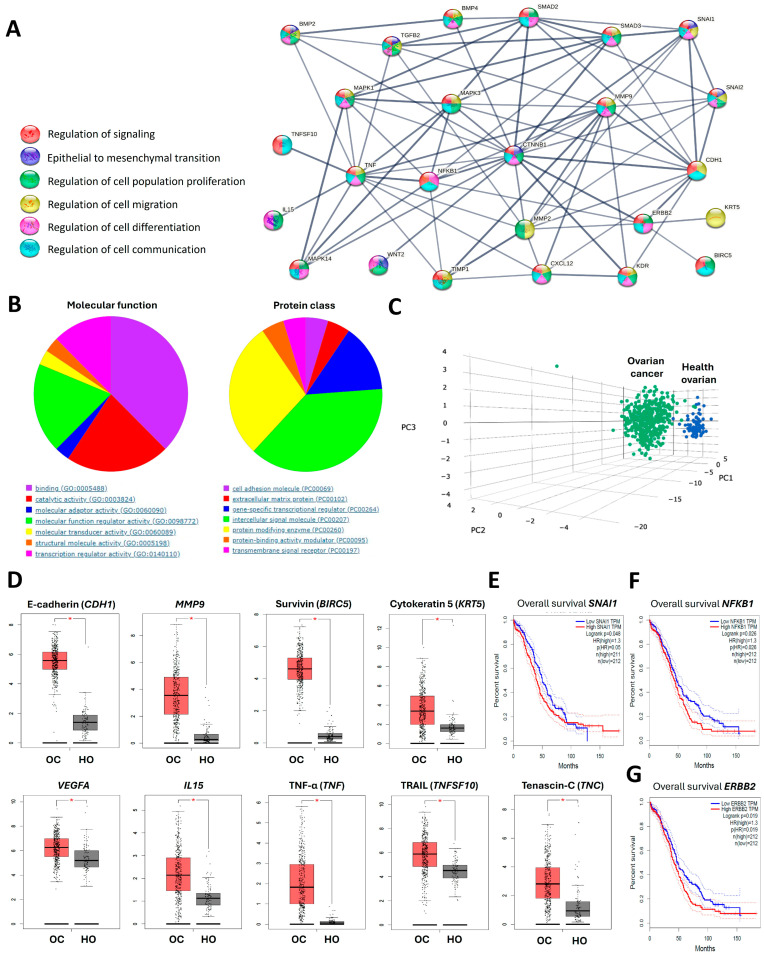
Functional enrichment analysis of targets modulated by the treatments with CM secretome and sEVs from ASCs and OC cells and overall survival of OC patients. (**A**) Protein–protein interaction (PPI) network illustrating the primary biological processes associated with the identified molecular targets (PPI enrichment *p*-value < 1.0 × 10^−16^). (**B**) Pie chart representing the most prominent molecular functions linked to these proteins and their class. (**C**) Principal component analysis (PCA) distinguishing OC patients and individuals with healthy ovarian tissue based on gene expression profiles of selected molecular targets (TCGA and GTEx datasets). (**D**) Gene expression profiles of E-cadherin (*CDH1*), *MMP9*, Survivin (*BIRC5*), Cytokeratin 5 (*KRT5*), VEGF-A (*VEGF-A*), IL-15 (*IL15*), TNF-α (*TNF*), TRAIL (*TNFSF10*), and Tenascin-C (*TNC*), comparing ovarian cancer (OC) patients to healthy ovary (HO) samples (|Log_2_FC|Cutoff: 1; *p* < 0.01; jitter size of 0.4; number of samples: 426 OC and 88 HO). (**E**–**G**) Kaplan–Meier survival curve illustrating the overall survival of OC patients, based on TCGA data, and stratified by gene expression levels (Cox regression analysis). * *p* < 0.05.

A protein–protein interaction (PPI) network was generated using all identified molecules, revealing 25 nodes with strong associations (high confidence) with key biological processes, including the epithelial-to-mesenchymal transition (EMT), regulation of signaling, regulation of cell population proliferation, regulation of cell communication, regulation of cell migration, and regulation of cell differentiation (Figure 8A).

Further analysis of the molecular functions of these targets indicated predominant roles in binding, regulatory, and catalytic activities, emphasizing the significant influence of the soluble mediators altered by the cells in the context of OC (Figure 8B). We also conducted a PANTHER analysis to classify the target proteins, revealing a predominance of intercellular signaling molecules, protein-modifying enzymes, and gene-specific transcriptional regulators (Figure 8B).

To investigate specific gene signature profiles associated with the analyzed molecules, we performed principal component analysis (PCA) (Figure 8C) to distinguish between TCGA samples (OC patients) and GTEx samples (healthy ovarian tissue). Among the 26 molecular targets involved in OC cell fitness, the expression levels of E-cadherin (*CDH1*), *MMP9*, Survivin (*BIRC5*), Cytokeratin 5 (*KTR5*), VEGF-A (*VEGF-A*), IL-15 (*IL15*), TNF-α (*TNF*), TRAIL (*TNFSF10*), and Tenascin-C (*TNC*) were significantly increased in OC samples compared to healthy ovarian tissue (Figure 8D).

Survival data of cancer patients from the TCGA dataset were analyzed using GEPIA to explore the prognostic significance of specific targets. A risk score correlating gene expression with OC prognosis identified that high expression levels of *SNAI1* (Figure 8E), *NFKB1* (Figure 8F), and *ERBB2* (Figure 8G) were associated with shorter overall survival in OC patients (hazard ratio [HR] = 1.3; log-rank *p*-value < 0.05) (Figure 8E,F).

## 4. Discussion

The interaction and communication between cancer cells and stromal cells are fundamental components of the tumor microenvironment (TME), critically contributing to the promotion of cancer cell proliferation, metastatic dissemination, and the acquisition of resistance to therapeutic agents. In this study, we investigated the role of extracellular signaling molecules in mediating the adaptation of recipient cells within the context of OC. Specifically, we analyzed the effects of in vitro exposure to the direct secretome present in the CM, as well as through co-culture systems enabling indirect communication between cell lines and targeted treatments involving sEVs independently of other soluble factors present in the CM. In these complementary assays, we exclude the effects of direct contact between ASCs and OC cells, which may represent conditions of early-stage tumors. This approach is based on the premise that, during early tumorigenesis, ASCs are likely influenced primarily by soluble factors and EVs released by tumor cells before direct cell–cell contact occurs. Within this investigative framework, we demonstrated that exposure to paracrine signaling from normal ASCs or OC cells consistently promotes tumor progression by activating a variety of pathways associated with cellular survival, proliferation, and metastatic spread. This includes molecular changes in ASC markers, reflecting a transition to a cancer-supportive phenotype that could prime the TME in initial disease stages. Consequently, we also identified that some of the overexpressed markers in this analysis are significantly associated with poorer clinical prognoses.

Herein, we show that ASC-derived sEVs significantly enhance the colony-forming efficiency of both OC cell lines after 8 days of culture with a concentration of 40 μg/mL. However, a decrease in proliferation rate was observed after 6 days of culture, particularly with the 10 μg/mL ASC-sEVs treatment. These findings suggest the presence of a dose- or time-dependent effect of ASC-derived sEVs on the expansion of OC cells. Notably, previous studies have also reported that ASC-sEVs suppress the proliferation of SKOV3 and other OC cell lines [24,25,26]. Interestingly, when the interaction between OC cells and ASCs was evaluated through indirect co-culture or treatment with ASC-derived CM secretome, the results consistently demonstrated that ASCs promote ovarian tumor growth [5,6,7,8,15,23,33]. Indeed, the bioactive molecules present in sEVs and CM secretome were shown to be different. Suzuki and colleagues [25] compared the miRNA profiles of small, medium, and large ASC-derived EVs, revealing a marked overexpression of *hsa-let-7b-5p* and *hsa-let-7e-5p* in small EVs, which was associated with their stronger antitumor activity. These findings highlight the complexity of ASC-derived factors and suggest that the distinct molecular cargo of sEVs may contribute to divergent effects on ovarian tumor biology, depending on the specific context and experimental conditions.

The dysregulation and mutation of key molecules within signaling pathways are critical drivers of cancer initiation and progression [34]. Tumor cells can achieve autonomous and uncontrolled proliferation by disrupting growth-promoting signaling cascades, with ASCs serving as pivotal sources of upstream signals. We demonstrate that a 6-day treatment with ASC-derived CM significantly increased the expression of TGFβII in both OC cell lines. This upregulation was accompanied by an increase in the expression of Smad 2/3 transducer proteins, indicative of the activation of the canonical TGFβ/Smad signaling pathway. Another receptor that mediates the phosphorylation of distinct Smads, BMP 2/4, was also upregulated in SKOV3 cells after 6 days of indirect co-culture with ASCs. Beyond canonical Smad signaling, ASC-derived extracellular mediators also activated TGF-β non-Smad signaling pathways, including the ERK and p38/MAPK cascades in OC cells. The significant expression of erbB2/HER-2 receptors in OVCAR3 cells further supports the involvement of MAPK signaling, as this pathway functions downstream of the receptor. A possible explanation for these outcomes is the significant levels of SDF-1α quantified in the CM secretome of ASCs. This chemokine activates the PI3K/Akt and MAPK/ERK signaling pathways, which promote cell growth and enhance resistance to apoptosis. Collectively, these findings suggest that ASCs, through the release of bioactive molecules in their secretome, contribute to establishing a microenvironment favorable to the progression and aggressiveness of OC. The activation of multiple signaling pathways linked to cell survival and proliferation reinforces the critical role of ASCs as potential mediators of tumor progression in OC.

The progression of epithelial-derived tumors to advanced stages relies on their ability to invade, metastasize, and undergo structural changes. We documented that ASC-derived sEVs and CM enhanced the invasive and migratory capacities of both OC cell lines in a dose-dependent manner. This increased motility was accompanied by the upregulation of NF-κB, β-catenin, and Wnt-2 expression. Wnt-2, together with the Tenascin-C glycoprotein, can activate the canonical Wnt/β-catenin signaling pathway, and collectively, these pathways can reprogram OC cells to proliferate and migrate [23,35]. Interestingly, we show that both ASC- and OVCAR3-derived CM contain high levels of Tenascin-C in their secretome.

The extracellular matrix (ECM), a natural barrier to tumor invasion and metastasis, is predominantly composed of collagen IV, which is extensively degraded by MMP enzymes whose expression is upregulated by transcription factors such as Snail [36]. Our results reveal a significant increase in Snail/Slug and MMP-9 expression in SKOV3 cells, aligning with previous studies that demonstrated enhanced migration of SKOV3 cells mediated by Snail and Slug [37] and MMP-9 expression in OC cells treated with ASC-derived CM [6]. The increase in MMP-9 enzymes can also be mediated by extracellular mediators present in the CM secretome. Specifically, both ASCs and OC cells enrich their secretome with MMP-2, TNF-α, and Tenascin-C molecules, which can act in autocrine or paracrine manners to enhance MMP synthesis, thereby favoring invasion and metastasis. However, the ASC-derived CM also exhibited higher levels of TIMP-1, a key inhibitor of MMPs, including MMP-9 [38]. Although previous studies have demonstrated a reduction in breast cancer cell migration and invasion following treatment with CM from immortalized MSC—an effect partially reversed by TIMP-1 inhibition [38]—the inhibitory activity of TIMP-1 may not be sufficient to counterbalance the heightened MMP activity observed in ASCs, which appears to be higher than in other MSCs sources [39]. These findings emphasize the complexity of ASC-derived factors in modulating ECM-related molecules, ultimately contributing to tissue remodeling and tumor progression.

Our results also show that both OC cell lines exhibited an increase in the expression of the epithelial marker E-cadherin after 6 days of treatment with ASC-derived CM. The existing literature suggests that enhanced E-cadherin expression occurs in the early stages of OC progression to avoid detachment-induced apoptosis and to give resistance to radiation and chemotherapy [40]. These findings seem contradictory since Snail and Slug, transcription factors known to repress E-cadherin expression, were also upregulated in OC cells and play an essential role in EMT. During this process, tumor cells undergo morphological changes toward a spindle-like phenotype and reorganize their cytoskeleton and cell–cell contacts, enabling individual cells or groups of cohesive cancer cells to escape the rigid epithelial organization. This transformation allows for increased motility and the ability to invade other tissues, which explains why EMT is implicated in cancer progression, metastasis, and drug resistance in various carcinomas. The implementation of the EMT program is a complex process and relies on a series of intracellular signaling networks involving signal-transducing proteins regulated by TGFβ-induced cascades, such as ERK, MAPK, Smads, as well as other pathways like Wnt/β-catenin and NF-κB signaling [41]. Notably, all these pathways were found to be upregulated following treatment with ASC-derived extracellular signaling molecules, such as TNF-α, which was quantified with the highest levels in the ASC secretome when compared to the CM of OC cells itself.

A series of distinct markers are used to define a cell as having either an epithelial or mesenchymal phenotype [41,42,43]. However, as observed here, colocalization of these two distinct sets of markers can occur in tumor cells, defining an intermediate EMT phenotype [42]. This suggests that OC cells undergo only partial progression through this process. On the other hand, tumor cells may retain and benefit from this binary framework, acquiring a hybrid epithelial/mesenchymal phenotype that was previously observed in OVCAR3 cells, positive for epithelial markers such as Krt18, β-catenin, E-cadherin, and Claudin-3, as well as for the mesenchymal marker Vimentin [44,45]. Consistent with these observations, our findings indicate that the increased expression of E-cadherin and Cytokeratin 5 in OC cells treated with ASC-derived CM, coupled with elevated levels of EMT transcription factors Snail/Slug and β-catenin, suggest the adoption of a hybrid epithelial/mesenchymal phenotype. This intermediate state, characterized by the coexistence of epithelial and mesenchymal traits, enhances tumor cell plasticity, thereby facilitating metastasis. Consistent with prior studies, this phenotype allows cells to balance adhesion and motility, supported by the activation of signaling pathways like TGFβ/Smad and MAPK, which contributes to the modulation of this hybrid state.

It has been postulated that ovarian carcinomas are heterogeneous tumors containing a subset of cells with stem cell-like properties, characterized by the expression of specific stem cell markers. Consistent with this, McLean and colleagues [46] described carcinoma-associated MSCs (CA-MSCs) exhibiting phenotypic markers similar to ASCs within OC tissue. These ASCs can be recruited into the TME, where they adopt a pro-tumorigenic phenotype mediated by the TGFβ signaling, thereby contributing to OC progression [13,14,15,20]. Therefore, understanding the mechanisms through which ASCs are co-opted by OC cells to acquire a tumor feature is crucial for developing targeted therapeutic interventions. Here, we clearly showed a dose-dependent effect of OC-derived sEVs on the proliferative and migratory capacities of ASCs. Interestingly, treatment with 40 μg/mL of sEVs from both OC cell lines led to a reduction in the proliferative and migratory capacities of ASCs compared to the 10 μg/mL dose. One possible explanation is that OC-derived sEVs, which contain pro-inflammatory or immunomodulatory factors—such as IL-15, TRAIL, TNF-α, and VEGF-A, as identified in the OC cell-derived secretome—may trigger inflammatory responses in ASCs. At higher concentrations, these responses could become detrimental, leading to cellular stress and diminished proliferative capacity. Further investigation into the metabolic profile of ASCs following treatment with different concentrations of OC-derived sEVs and CM could provide valuable insights into the mechanism underlying these effects.

Since tumors consist of cells with different invasive potential, the release of extracellular signaling molecules may serve as a mechanism to transfer invasive and migratory capacity from one cell to another, thereby driving tumor progression. Herein, ASCs treated with OC-derived CM secretome or sEVs from both cell lines increased their invasiveness and migratory capacities after 24 h of culture. This enhanced motility is associated with the significant expression of MMP-9 and Wnt-2/β-catenin signaling, which act synergistically to facilitate the degradation of the ECM, enabling malignant cells to invade adjacent tissues and migrate to distant sites [34]. Interestingly, ASCs treated with SKOV3-derived CM exhibited increased expression of E-cadherin, Snail/Slug, and β-catenin, whereas treatment with OVCAR3-derived secretome led to the upregulation of only Cytokeratin 5 and β-catenin. These findings suggest that ASCs display a cell type-dependent response to tumor-derived secretomes. In fact, OVCAR3 and SKOV3 cells differed in the levels of extracellular signaling molecules, as quantified by the multiplex immunoassay. Specifically, the OVCAR3 secretome contained higher levels of TNF-α, Tenascin-C, and TIMP-1, while SKOV3 exhibited elevated levels of the TRAIL cytokine. These differences in the secreted factors profiles of the OC cell lines may have influenced the variations in the expression of ASC markers observed.

Notably, treatment with SKOV3-derived CM also elevated the expression of TGFβII and components of both the canonical (Smad 2/3) and non-canonical (ERK 1/2 and MAPK) TGFβ signaling pathways. As documented in a proteomic study by Sharma and colleagues [19], these cell source-dependent differences may be attributed to the distinct protein profile uniquely present in SKOV3-derived exosomes, which is absent in the EVs from OVCAR3 cells. Apart from this, both OC cell lines induced a marked increase in the expression of the BMP 2/4 receptor pathway and the oncogene erbB2/HER-2 protein, emphasizing the diverse and robust signaling mechanisms activated within ASCs that enable them to acquire a tumor-supportive role in the microenvironment.

As previously discussed, the hybrid epithelial/mesenchymal state is typically characterized by the co-expression of both epithelial and mesenchymal protein markers [47]. This hybrid state represents an intermediate stage in the transition toward a mesenchymal phenotype, a process that remains reversible as tumor cells undergo mesenchymal-to-epithelial transition (MET) [48]. As progenitor mesenchymal cells, ASCs inherently possess phenotypic features commonly induced by EMT in epithelial cells and, therefore, do not need to undergo EMT to acquire these characteristics. However, because EMT and MET are a dynamic and gradual process, stem cells such as ASCs can undergo phenotypic changes that mirror these transitions [43]. In the case of MSCs, this hybrid state can be described as a “metastable” phenotype, characterized by the simultaneous presence of epithelial and mesenchymal traits alongside stem cell properties. Within this metastable phenotype, MSCs gain the ability to metastasize, thereby contributing to cancer progression [43,49].

Such a metastable phenotype may enable individual tumor cells to shift toward either a more epithelial or mesenchymal differentiated state. The identification of OC stem cells with self-renewal capabilities and high epithelial plasticity raises the intriguing possibility that these cells possess greater metastatic efficiency, potentially accounting for the majority of metastasizing OC cells [40]. This phenomenon was previously described in an OC model by Fan and colleagues [50]. Mechanistically, MSCs can undergo epigenetic reprogramming to adopt a partial MET phenotype when exposed to direct or indirect co-culture with OC cells. These CA-MSCs were characterized by enhancer-enriched DNA hypermethylation, altered chromatin accessibility, and the acquisition of repressive histone marks favoring migration, invasion, colonization, and cell adhesion pathways, playing a critical role in establishing a new metastatic TME [50].

Molecular intratumor heterogeneity can drive highly aggressive clinical behavior by enabling the tumor to exploit both EMT- and MET-related processes within its cells [40,43]. Supporting this, OC patients treated with neoadjuvant therapy who exhibited a normal-like MSC phenotype, characterized by a normal MSC DNA methylation profile, demonstrated significantly improved progression-free survival (PFS) of 870 days, compared to only 266 days in the CA-MSCs group [50]. We further examined the relationship between molecular targets influenced by bioactive molecules secreted by ASCs and OC cells using an in silico functional enrichment analysis to evaluate the prognosis of OC patients. The analysis revealed that altered proteins and secreted factors are strongly associated with EMT, cell signaling, proliferation, migration, communication, and differentiation. All these processes are intricately linked to the complexity of TME and are known to be regulated by the TGFβ pathway, which operates through autocrine or paracrine mechanisms to influence cell fate [14,50]. Altered molecular targets, such as E-cadherin (*CDH1*), *MMP9*, Survivin (*BIRC5*), Cytokeratin 5 (*KRT5*), *VEGFA*, *IL15*, TNF-α (*TNF*), TRAIL (*TNFSF10*), and Tenascin-C (*TNC*), exhibited increased expression in our study, which correlates with their upregulation in tumor samples from individuals with OC. Additionally, *SNAI1*, *NFKB1*, and the oncogene *ERBB2*—associated, respectively, with the EMT/MET phenotype, regulation of pro-migratory and anti-apoptotic genes, and cell survival—were strongly linked to significantly poorer overall survival in OC patients. Therefore, identifying novel clinical prognostic biomarkers and therapeutic targets is essential for improving the survival outcomes of patients diagnosed with OC.

It is also important to acknowledge the limitations of our study. Although we qualitatively assume an equal distribution and internalization of sEVs from different cell sources, we did not perform a quantitative assessment of their uptake. The inclusion of a flow cytometry-based quantification could provide a more precise evaluation of potential variations in vesicle uptake. Additionally, since we worked with a pooled sEVs sample from different selected donors despite the strict criteria, we must highlight the potential impact of donor heterogeneity. Based on this, we suggest that future studies investigate donor-specific differences in sEVs composition and function. Furthermore, given that we observed cell line-specific responses in OC cells, the inclusion of analog cell models, which were absent in this study, could be valuable for validating our findings. Another limitation of this study concerns the molecular composition of sEVs. A detailed molecular characterization, such as proteomic or transcriptomic profiling of sEVs, would help determine whether the bioactive components of sEVs contribute to the differential effects observed between ASCs and OC cell lines. While CM- and sEV-based approaches allow for the study of unidirectional effects, they inherently present limitations. The inclusion of direct co-culture systems, which were not employed in this study, could better represent the bidirectional communication between the investigated cell types. Additionally, our in silico analysis was based on the TCGA dataset, which includes patient samples from ovarian serous cystadenocarcinoma. Analyzing multiple OC subtypes would provide a more comprehensive understanding of ASC–tumor interactions. Finally, our results did not differentiate between endogenous sEVs secreted by the cells and those exogenously added in the sEV-based experiments. We acknowledge this limitation and suggest the use of previously labeled sEVs or the quantification of sEVs secreted by cells prior to the addition of exogenous vesicles in future studies.

In addition, based on our results, we open the field for future research that focuses on elucidating the direct and indirect interactions between OC cells and ASCs and in the identification of key mediators driving the pro-tumorigenic transformation of ASCs. Also, investigating the long-term effects of sEVs and secretome exposure on ASCs and OC cells beyond the 6-day treatment window could provide deeper insights into tumor progression and metastasis. Exploring the functional role of the hybrid epithelial/mesenchymal phenotype in metastasis, chemoresistance, and immune evasion seems to be crucial. Developing therapeutic strategies to disrupt EVs-mediated communication or paracrine signaling, such as small molecule inhibitors or EVs-based therapeutics, could improve treatment efficacy. By addressing these directions, we can advance our understanding of tumor–stroma/stem cell interaction and develop innovative approaches to combat OC.

## 5. Conclusions

The current findings highlight the critical role of tumor-derived extracellular signaling molecules, including sEVs and cell secretome, in driving a pro-tumorigenic phenotype in ASCs, thereby contributing to the aggressive behavior of ovarian carcinoma. The observed co-expression of epithelial and mesenchymal markers in ASCs and OC cells suggests a hybrid epithelial/mesenchymal phenotype, which enhances cellular plasticity, tumor progression, and metastatic potential. This dynamic interaction, mediated by soluble factors and sEVs, underscores the intricate bidirectional communication between stromal and cancer cells, wherein both cellular sources of extracellular mediators influence each other to alter the behavior of the TME in favor of metastasis. Notably, tumor-associated MSCs emerge as critical players, secreting paracrine factors that drive tumor growth and metastatic spread, ultimately correlating with poor prognostic outcomes. These insights provide a more comprehensive understanding of the molecular mechanisms underlying OC progression and suggest that targeting the crosstalk between ASCs and tumor cells, particularly through tumor-derived sEVs, represents a promising therapeutic strategy to improve the effectiveness of conventional cancer treatments and mitigate tumor aggressiveness.

## Figures and Tables

**Figure A1:**
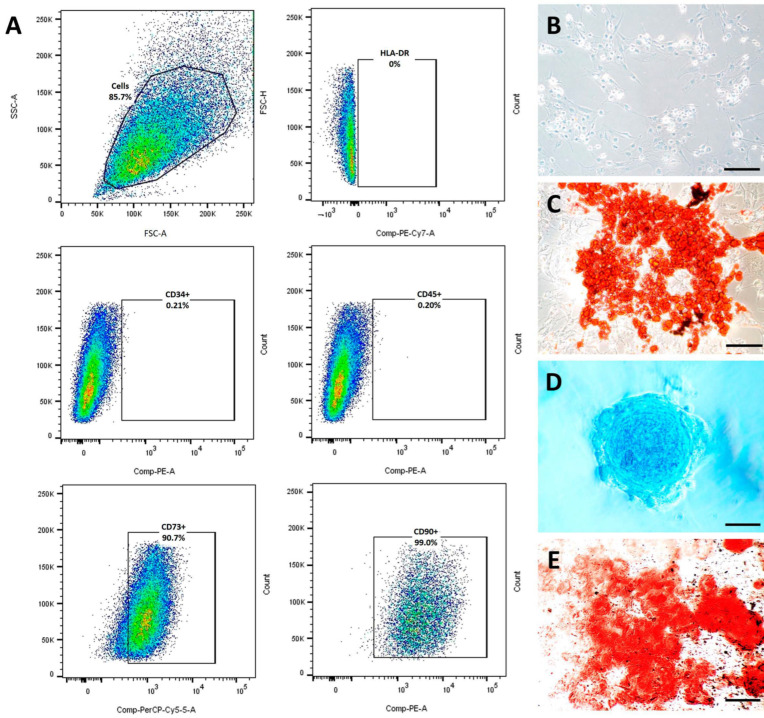
Characterization and differentiation of ASCs. (**A**) Flow cytometry of the pool of ASCs in the 3rd passage isolated from female donors undergoing abdominoplasty surgery. ASCs were negative for CD34, CD45, and HLA-DR (<1% of total cells) and positive for surface markers CD73 and CD90 (>90% of total cells). Entries were defined according to isotype control. (**B**–**E**) Representative images of ASCs subjected to in vitro differentiation at the 3rd passage. (**B**) Control (no modifications); (**C**) adipogenic differentiation in red (lipidic vacuoles); (**D**) chondrogenic differentiation in blue (proteoglycans); and (**E**) osteogenic differentiation in orange/red (calcified matrix). Scale bars = 50 µm (**D**,**E**), 100 µm (**B**,**C**).

## Data Availability

Data are contained within the article.

## Abbreviations

The following abbreviations are used in this manuscript:

ASCs	Adipose-derived mesenchymal stem cells
ASC-sEVs	Small extracellular vesicles derived from adipose-derived mesenchymal stem cells
BMP2/4	Bone morphogenetic protein—2/4
CA-MSCs	Carcinoma-associated mesenchymal stem cells
CM	Conditioned medium
CFDA-SE	Carboxyfluorescein diacetate succinimidyl ester
CFSE-sEVs	Small extracellular vesicles stained with carboxyfluorescein diacetate succinimidyl ester
DMEM/F12	Dulbecco’s Modified Eagle’s medium
ECM	Extracellular matrix
EMT	Epithelial-to-mesenchymal transition
EVs	Extracellular vesicles
IOP	Optical densitometry index
ISEV	International Society for Extracellular Vesicle
MAPK	Mitogen-Activated Protein Kinase
MET	Mesenchymal-to-epithelial transition
MMP	Matrix metalloproteinases
MSCs	Mesenchymal stem cells
MTT	3-(4,5-dimethylthiazol-2-yl)-2,5-diphenyltetrazolium bromide
NF-κβ	Nuclear transcription factor kappa B
NTA	Nanoparticle tracking analysis
OC	Ovarian cancer
OVCAR3-sEVs	Small extracellular vesicles derived from OVCAR3 cells
PBS	Phosphate-buffered saline
PI	Protease inhibitor
PMSF	Phenylmethylsulphonyl fluoride
RT	Room temperature
SEC	Size exclusion chromatography
sEVs	Small extracellular vesicles
SEM	Standard error of the mean
SKOV3-sEVs	Small extracellular vesicles derived from SKOV3 cells
TEM	Transmission electron microscopy
TGFβII	Transforming growth factor beta 2
TME	Tumor microenvironment
Wnt-2	Wingless-type MMTV integration site family

## Appendix A

### Appendix A.1. ASCs from Female Donors Were Successfully Characterized as MSCs

Before the experiments, ASCs isolated from female subjects undergoing abdominoplasty surgery were successfully characterized as MSCs following the guidelines presented by the International Society for Cellular Therapy (ISCT) on the minimal criteria for defining MSCs [51] (Figure A1). Our ASCs were positive for surface markers CD73 and CD90 and negative for HLA-DR, CD34, and CD45 (Figure A1A). Furthermore, ASCs demonstrated their potential for differentiation into mesenchymal derivates under in vitro conditions, such as adipocytes by the presence of lipid vacuoles in the cytoplasm, chondrocytes by the synthesis of proteoglycans, and osteocytes by the presence of calcified matrix (Figure A1B–E).
